# Some Diseases Incidental to Pregnancy and Parturition in the Cow

**Published:** 1902-10

**Authors:** A. M. Crichton


### SOME DISEASES INCIDENTAL TO PREGNANCY AND PARTURITION IN THE COW.

By A. M. Crichton, M.R.C.V.S.

Parturient Eclampsia. *Definition.* An epileptic and convulsive affection affecting the cerebro-spinal nervous system, accompanied by tonic and clonic spasms of the muscles of various regions of the body. It occurs in cows both before and after parturition.

*Causes.* Opinions differ in regard to the causes. By some it is supposed to be due to the retention of urinary elements in the blood; by others to hyperæmia of the brain, and by others still to reflex irritation of the nervous system, which brings about renal insufficiency and albuminuria; and exposure to cold winds and wet may bring on the disease.

*Symptoms.* The cow takes suddenly ill by foaming at the mouth, champing the jaws; full, wild-staring eyes, excited expression of countenance, head turned to the side, sometimes pulling back on the chain, licking at the foreleg, edge of the feeding-trough, or anywhere convenient; some cows bellow, others do not; twitching of the muscles of the body and limbs; sometimes difficulty of respiration; sometimes the cow becomes comatose. Most cases occur after calving; those which occur before are generally about mid-term, and in heifers. The attacks after calving generally occur from eight to sixteen days afterward. The disease occurs in cows of all ages, and generally in cows of lean condition. The reverse of this happens in parturient apoplexy, as it is a fat cow and a good milker that goes down with it. The most of cases do not get off their feet, although I have had them that did and lay for a night before getting up again. It might be possible to confound this disease with parturient apoplexy, but if we are careful to note the symptoms it should not happen. I will endeavor to point out the essential differences between the symptoms of the one and the other. There are about eight prominent symptoms that we should bear in mind when we are called in to see cases of this description. I will enumerate them as follows:

PARTURIENT APOPLEXY. It mostly occurs in cows at third calving and afterward; it never occurs before calving, and generally within forty-eight hours thereof. One attack predisposes to another one; most common in fat cows; patient fails to keep standing; rapidly followed by coma; earliest period of recovery is generally eight to sixteen hours; prognosis generally very unfavorable till Schmidt treatment found out; now more favorable.

PARTURIENT ECLAMPSIA. Occurs in cows of all ages, but mostly at first and second calving; occurs in cows before calving; if after calving, hardly ever before the eighth to sixteenth days; one attack not necessarily succeeded by another at next calving; most common in lean cows; patient mostly can stand through attack; the cow only occasionally becomes comatose; recovery takes place in a few hours generally; prognosis favorable and the treatment successful.

Now, gentlemen, these are the principal features in contrasting these two diseases, and I will now give you the most successful treatment that I have found for this disease after trying several remedies.

*Treatment.* First get the cow made comfortable in a good warm box or byre free from draughts, and as the morbid condition of the nervous system may be brought about by cold winds, wet, or draught, it is necessary to do this most needful part of the treatment; then, if the bowels are correct, I give no laxative, but prescribe from ʒiss to ʒij ext. belladonna every three hours as long as necessary. Bleeding may be resorted to if the foregoing treatment fails, but this is not usually the case. If the kidneys do not act promptly and sufficiently I prescribe ℥ij spt. ether nit. in a little cold water or along with the ext. belladonna; give about two quarts of linseed tea every few hours if the animal will have it, and this is usually all that is necessary for the treatment.

ABORTION. *Definition.* The expulsion of the fœtus and the fœtal membranes before the natural period. Abortion may be considered under two heads—non-contagious and contagious—and I will just consider the sporadic or non-contagious form. The normal period of utero-gestation in the cow is about nine-and-a-half months, some more, some less, and an abortion usually takes place from the third to the seventh or eighth month. After that month it may be termed a premature birth.

*Causes.* These are very numerous, and they may be divided into internal and external. Of the internal causes the general health of the cow is of the greatest importance. Any contagious or febrile disease in the system of the mother is a cause or factor in bringing about abortion, such as tuberculosis, pneumonia, indigestion, gastric tympany, excitement, nervousness, ergotized food, and, of course, the infecting bacillus.

*External Causes.* Cold and wet, changes of temperature, thunder and lightning, frights, narrow doorways, pressure over the uterus, overexertion, foul smells, ice-cold water, too much food, putrid, stagnant water, sewage, frosted turnips, potatoes, or roots of any kind, or rotten roots, mouldy, bad hay, etc.

I now come to the contagious form of abortion, and in giving the treatment for the sporadic variety I fancy it would be better to consider the treatment of both together, which I intend doing. This is by far the most serious disease of the two under consideration, and it is due to a special bacillus, which I believe was first demonstrated in the year 1895 by Professor Bang, he having made a series of post-mortems of cases, and also made several examinations microscopically of the liquid between the walls of the uterus and the envelopes of the fœtus. In cases that showed premonitory symptoms of abortion he had the cow killed and the experiments carried out as before mentioned, and in every case that he examined this liquid from the womb of the affected cow he could demonstrate the presence of this bacillus in great numbers, and they were unmixed with any other organisms; so, gentlemen, this surely places the theory of contagion beyond doubt. Professor Bang also cultivated this bacillus outside the animal body. He found that it was not easily cultivated by the ordinary methods, but he succeeded in growing it in blood serum rendered solid by the addition of gelatin and agar, and when it was grown in this medium it exhibited quite remarkable characters, absolutely distinguishing it from any other known pathogenic organism. In subsequent cases Professor Bang demonstrated the same bacillus in the discharges from the uterus or vagina of cows that had aborted. So you see, gentlemen, that contagious abortion is really a disease due to a specific organism. Another way in which its contagiousness has been demonstrated is by taking part of the discharge from an abortive cow and putting it into the genital passages of a pregnant cow, and in nearly every case the cow will abort some time afterward. I also believe that this bacillus is able to multiply outside the animal body, even in soil, dirt, or manure, and this may go a great length to demonstrate the first outbreak in a herd that was previously free from this disease, and it is sometimes very difficult to know where the contagion comes from in the first case in a herd of cows; but in reality it comes from some source—from an apparently healthy, newly bought cow, or from the land they are grazing on having been previously grazed by some infected cow, or from feeding stuff or fodder imported or brought to the byre from an infected place. There is always a cause for everything that happens in this life.

This bacillus is supposed to gain an entrance by the vulva into the womb or become absorbed through the mucous lining of the vulva into the circulation (because abortion can be produced by injecting the bacillus hypodermically); it then gets into the circulation, locates itself in its habitat, so to speak, and sets up the symptoms that we see terminate in the expulsion of the fœtus and membranes prematurely.

*Causes.* The primary cause is, of course, the bacillus. How does it get there? It is sometimes due to an infected bull during copulation; sometimes to a newly purchased cow brought from a tainted herd. This will bring it into a byre that was never known to have a previous case, and generally when one cow aborts there are several if not all in the same house. One reason given for this is the contagious nature of the disease, and if the cow retains the after-birth there is always a discharge of infected material, and this soils the tail and thighs, and by means of the tail the infective material is switched about to the other cows in the same house. It may also be disseminated by the discharges into the gutters running past the other cows. By inserting infective material into the genital passages of a healthy pregnant cow you will generally have abortion in from fifteen to thirty days. As a rule, the cow's health is not much affected; it is purely and simply a disease of an infective nature that affects the vagina and womb, fœtus, and fœtal membranes of pregnant cows, mares, and ewes.

*Prevention and Treatment.* I intend to make this as short as possible. To consider measures of prevention we must think about what will keep a stock in health as well as to the measures that are necessary to bring an outbreak to a speedy termination. With regard to the former, every person that buys a cow is exposed to getting his herd infected. When buying a new cow isolation for a period is a safe plan. On buying a new stud bull isolate for a time, and disinfect penis and genitals for some time before using him for stud purposes. There is a big risk in keeping a bull for the service of outside cows; he should be disinfected and washed after each service.

To bring an outbreak of this disease to a speedy end there are a great many difficulties which present themselves to us. The first is that for weeks and probably for months a cow may be affected and capable of infecting other cows without presenting any symptoms pointing to her dangerous state. It, therefore, often happens that before the existence of the disease in the herd is suspected and realized a lot of the cows become affected. A cow is infected before she has aborted, but she is more dangerous after and for several weeks, as she then liberates an army of bacilli capable of infecting all the other cows in the herd. When she aborts it is a good plan to burn the fœtus and membranes. If this cannot be done bury them away from access to cows, and disinfect them before burial, and over the grave put some quick-lime.

The cow-house will require to be disinfected often and thoroughly, and the cows therein will also require to have the vagina washed out, and the tail and thighs, udder, and even up the sides, over the back, etc.; in fact, as far up as the tail reaches, well sponged or brushed with a reliable antiseptic to destroy the bacilli, and the more thoroughly and frequently this is done the better the chance of eradicating the disease. There are various agents that may be used for this purpose. Some taint the milk and are not practicable. Sulphate of copper is good to use, and does not taint the milk—six ounces to one gallon of soft water; also sprinkle the floor and gangway with powdered lime; and as a further precaution the outer genitals, root of the tail, and, in fact, the whole tail, it is well to sponge once a day with a solution of Jeyes' carbolic or hydrarg. perchlor.: hyd. perchlor., ʒijss; acid hydrochlor., ℥ijss; aqua, 2 gallons. This solution is dangerous, and any of it that is left should be buried; but it cannot be prepared in metallic vessels, for it corrodes them. This disease in its treatment involves a great deal of trouble and labor. When the number of cows that have aborted are many, and there is no chance of isolating them, the best plan is to put the aborted cows at the lower end of the house, so that the urine does not run past the healthy ones; this will reduce the risk of infection. If a cow aborts this year it does not follow that she will abort next year; in fact, experience shows that it is the exception, not the rule.

In regard to the manure, is it not possible that the abortion bacillus will retain its vitality for a length of time in it? I would recommend in this case that the manure be mixed with a sufficiency of lime and left for a good long time before being put onto land that cows will be grazed on afterward. I must say that the wooden cow-houses, when they get rotten, and without paved gutters; the want of cleanliness, houses badly lighted, badly ventilated, badly drained, and in some cases not drained at all, all militate against the veterinary surgeon in stamping out any contagious disease in this country.

INVERSION OF THE UTERUS. This is a very serious condition in any female animal.

*Causes.* Retention of fœtal membranes, irritation of mucous membranes of the organ at the time of parturition, wounds, scraps, hard calving, injuries during calving, overfeeding, tympany, indigestion, ice-cold water to drink after calving.

*Treatment.* Now, when the cow does put out her uterus, the first thing to do is to get to the case as soon as possible after you have been notified about it. Some farmers know how to wrap it up in a damp, hot blanket and send at once for the veterinary surgeon, and when he arrives the first thing he should do is to remove the cleansing, if necessary. Cleanse the organ, dry it with a cloth, and put some antiseptic and soothing dressing upon it, preferably an oily preparation, and return it by gentle manipulation, and put on a truss or West's uterine clamp, not screwed up so tightly as to prevent the animal from urinating or to hurt the vulva. The cow generally strains a good deal after this. To combat this we give some anodyne mixture, chloral hydrate, chlorodyne. Raise the hind-quarters with freshly-cut sods. There is some danger of metritis, so a little tr. aconite and a mild laxative will prove very beneficial.

RETENTION OF FŒTAL MEMBRANES. SEPTICÆMIA. This disease is due generally to a cow calving before the natural period, or from debility, weakness, want of blood, and it mostly happens in hot weather. Dealers' cows that are "faired" and "trained" are very liable to retain the cleansing, and so are very liable to have septic poisoning.

*Causes.* The anatomical arrangement of the uterus in the cow, difficult parturition, abortion. Aged cows retain the membranes longer than young ones, as a rule. Contraction of the cervix uteri will prevent expulsion of the fœtal membranes. Cows driven long distances to fairs and trained long distances produce fatigue and reduce the general health of the animal.

*Symptoms.* Usually part of the membranes protruding, but if not there is a certain amount of shifting of the feet, uneasiness, urinating, and passing feces often; then there is twitching and curling of the tail. One very prominent and reliable symptom is that the tail is carried farther off from the body than usual, in a kind of an arched manner. If putrefaction has commenced there will be a dirty, sanious, offensive discharge from the vulva, mingled with shreds of putrid membrane. At this stage there is generally constitutional disturbance; the animal is dull, dejected, appetite lost or bad, respirations hurried, temperature elevated, secretions suspended. The complications that follow this state of affairs are generally septicæmia, metritis, metroperitonitis, vaginitis, leucorrhœa, etc.

*Treatment.* If the membranes are not expelled in three or four days or less they are better removed, and the uterus irrigated with a solution of ozophene, chinosol, or suitable disinfectant. There are various preparations that can be used for this purpose—carbolic acid, chinosol, ozophene, Jeyes' fluid, potassium permanganate. I find about two irrigations sufficient. If the cow strains badly I give anodynes. If in weak health and of low condition, tonic and external antiseptics are indicated. If septicæmia is feared, give plenty of external antiseptics, with tonics, stimulants, and good food, easy of digestion and assimilation, and try to keep the animal's strength with milk, eggs, gruel, etc. Sulphites, hyposulphites, sulphocarbolites, quinine, hydronaphthol are indicated; and to prevent infection of healthy subjects the patients should be isolated, excreta promptly burned, and covers, etc., used by them disinfected before being used with other cattle.

INVERSION OF THE VAGINA, or "showing of the reed," as the farmers call it in this district, is often seen in cattle. The causes are injuries, rupture of the internal bands that hold the organ *in situ*, constipation, protracted parturition, fatigue from long travelling after parturition, abortion, placental retention. Old cows when heavy in calf show it often. Low stand behind, and too much fall for the stand, so that when the cow lies down the whole weight of the calf, stomach, bowels, etc., are thrown back on the os uteri, and this tends to relax passages and to force out the vagina.

*Symptoms.* Generally makes its appearance when the animal is lying, and mostly seen in pregnant cows. The vagina differs in appearance from the uterus, as there are no cotyledons on it; and it is also of less volume, with a small, round orifice in the centre.

*Treatment.* Cleanse and return it after dressing with a little ol. carbolic. This is not a very dangerous affection, although it is sometimes very troublesome. I had a case here a short time ago; this was a prize Kerry cow, and she wore a truss for three months before calving, and she calved here a few weeks ago, but did not cleanse. The os contracted very shortly after the calving, and I could not remove the cleansing, but I irrigated the uterus with a solution of osophene, 1 : 30, and she has done well.

LEUCORRHŒA, commonly known as "the whites," is a chronic discharge from the vagina and womb, of a white, flaky color; sometimes inodorous, at other times of a rusty color, and mucopurulent and very offensive.

*Causes.* Debility, difficult parturition, uterine excitement, polypus, prolonged irritation of the genital passages, and as a sequelæ to tuberculosis. Sometimes the bull gets balanitis, and gives the cow he serves the disease, and sometimes she aborts.

*Symptoms.* Discharge from vulva, which is generally pale, bloodless, and flaccid. In other cases where the discharge is flaccid the mucous membrane is rough and reddened and thickened, with stricture of the vagina as an accompaniment. Animals so affected are frequently in œstrum, but very uncertain as to breeding, and if they do become pregnant they very often abort. Some constitutional disturbance is usually present, the milk diminished, the appetite very indifferent, and the animal fails in condition.

*Treatment.* In the primary stages cleanliness, frequent injections of tepid water, with weak solutions of alum, zinc sulphate, and potassium permanganate; the administration of a saline laxative, followed by tonics and laxative diet. In secondary or chronic cases injections of tannic acid, ferri sulphate, boric acid, iodoform, or, if the discharge is offensive, acid carbolic, 1 : 40, or ozophene, 1 : 30, may be used. Internally, mineral tonics, iron or copper, for preference, with quinine, with generous laxative, nutritious diet, will generally result in a cure.

METRITIS AND METROPERITONITIS. *Definition.* Inflammation of the uterus, inflammation of uterus and peritoneum. I shall consider these diseases under two heads: 1. Simple metritis is the simple inflammation of the organ. 2. Septic metritis, inflammatory action due to the presence of a bacillus in the uterus and in the discharges therefrom.

*Causes.* Unskilful and rough usage at calving, injuries, colds, draughts, retention of placental membranes, external violence, septic infection.

*Symptoms.* Usually ushered in with rigors, pain on pressure over region of womb. Tumefaction of vulva, with heat and dryness; then follows a discharge of purulent, offensive matter from the vulva, increase of temperature, quick, small pulse, increased respirations, grinding of the teeth, secretion of milk suspended; bowels constipated; on passing feces animal evinces pain. On examination per vaginam the os uteri will be found hot and sensitive. Sometimes discoloration is seen on examination per vaginam.

*Treatment.* Explore vagina if possible and ascertain if fœtal membranes are away. There may be some shreds left which may be putrefying in the womb. If so, wash out with a reliable disinfectant and antiseptic, give a mild laxative with anodynes, and apply hot-water blankets around the abdomen and see to the general comfort of the animal. If we fear the metritis is of a septic origin try the administration of internal antiseptics and stimulants, with nutritious diet and thorough irrigation of the uterus and vagina every day at least with suitable antiseptic solution. Some recommend packing the womb, but I have never resorted to this in cattle practice.

Polypus in the Vagina. These are frequent in the cow, attached to the mucous membrane a little distance from the orifice. The shape is generally that of a pear. I removed one weighing four and one-quarter pounds from the vagina of a cow lately by ligature and excision, and used a simple antiseptic dressing for a few days. The cow did all right.

Parturition. Now, gentlemen, I just wish to say a few words in regard to parturition in the cow. As a rule, the messenger that comes for us in these cases is sweating and out of breath, and will gasp out that Mr. So and So wants you to come to a cow that cannot calve. After asking a few questions we pack up a few necessary instruments, ropes, and the usual paraphernalia, and proceed with all speed to the scene of action. When we arrive there and begin to explore the case we generally find that Mr. Handyman has been there before us, and has spoiled the case and left it, because he could not do it. There are just a few positions that I wish to say a few words about. 1. Breech presentation. 2. One fore and one hind limb. 3. Two forelimbs, with head away back. 4. Twist of neck of womb.

In breech presentation push back buttocks of calf right as far up and back as you can, then get hold of foot and turn sideways and upward, and you will get it into passage quite easily. Do likewise with the other foot, and the calf generally comes quite easily.

In one fore and one hind you require to be able to judge which way the fœtus will come away easiest. Generally to push back the forelimb is safe practice, as by doing so you have no trouble to look for the head; then get up the other hind limb, and the fœtus comes away with little trouble.

In the case with the two forelegs with the head back the position is generally the result of long-continued straining of the cow without delivery. If the head cannot be reached by the hand try a blunt hook, and, if unsuccessful, then try and remove one forelimb, which is sometimes a very difficult job. You should then be able to reach the head and get the fœtus away. Then, as regards twist or torsion of the womb, this generally requires the cow to be thrown and rotated from one side to the other until the twist is undone, and then delivery is usually easily enough accomplished. Some veterinary surgeons recommend jamming the fœtus in passages and take it away in that manner. After-treatment is anodynes if necessary, and fomentation if needful.

Parturient Apoplexy. This disease used to be very fatal in the cow until Herr Schmidt, of Kolding, found out the potassium iodide treatment. There are a good many theories put forward as to the cause, such as clean milking immediately after calving; by ptomaines, the products of decomposition of food in the stomach and intestines (but I look upon this as the result, not the cause, of the disease); by anæmia of the brain, and by auto-intoxication. I favor this view, and believe milk-fever is due to some toxic material that gains entrance or is manufactured in the animal economy, and thus gaining access to the blood-stream. It may be due to some bacillus, but for that I cannot say. By some it is supposed to arise from the liver not being able to cope with the waste products in the blood during the time of rapidly rising lactation, especially as the liver and, in fact, all the glands in the animal economy have been in a state of comparative quiescence during the dry period. The light food the animal gets when dry is suddenly altered to stimulating food, and this condition may give rise to the auto-intoxication that produces the symptoms that we all so well know; and the eight principal symptoms have been put before you when contrasting them with puerperal eclampsia, so I will go on to the treatment and sequelæ. This much, then, can be said of milk-fever, that it may be considered a genuine auto-intoxication caused by the accumulation or manufacture in the system of toxic material during the first stages of lactation. This circulating through the blood causes the symptoms of poisoning of the whole system of the animal. In relation to this theory I would point out that only good milkers are attacked, and that those which have been dry for some time are more liable, especially those which have been fed on rich food when dry, and cows that are hardly ever outside the byre. These sort of cattle, after an easy calving, go at the milking in a hurry (as we may say), and arrive at the height of lactation very quickly, and in this way the intoxication occurs, owing to the overburdening of the liver and kidneys with work. Just as when the air we breathe is polluted with a certain amount of $CO_2$ we show the effects of $CO_2$ poisoning, so when the toxic material gets to a certain percentage in the blood of the cow she shows the symptoms of poisoning. Of the percentage that it takes I cannot say, but this is the theory I hold in regard to milk-fever, and upon this I base my treatment; and whether it is a product of casein, nuclein, albumin, or what, I am not prepared to say. Clearly it is the province of our scientists to elucidate such problems, for the general practitioners have neither time, money, nor appliances to do so.

*Treatment.* I have tried a number of remedies in milk-fever. I have tried the following injections into the udder, and they have proved fairly efficacious: chinosol, from 40 to 60 grains, according to the size and weight of the cow; boiled water, 2 quarts; and after milking the cow clean and disinfecting the udder and teats, when about the blood-heat I inject one-fourth into each teat. I also give the following every three hours alternately if the cow can swallow, and if in a weak state I give 4 ounces of spt. ammon. arom. in 1 pint of cold water, and if she cannot swallow I inject with the stomach-pump: Liq. ammon. acet. conc. (1 in 7), ʒij; spt. ether nitrosa, ℥ij; aqua, ℥xij; then whiskey, ℥iij, or spt. ammon. arom., ℥iij; aqua, ℥xij. I sometimes use the following injection, which also is very good: Iodine resub., 4 grs.; pot. iodide, 4 to 5 drs.; boiled water, 2 quarts, to be used as the foregoing. The drenching part of the treatment must be done with great care.

The sequelæ are: mechanical bronchitis from regurgitation of food, mammitis, metroperitonitis, from giving drastic purgatives; pneumonia, bruised knees and hocks, fatty degeneration of the muscles of the thighs from bruises, paralysis, choking at drenching. If a cow has tuberculosis in a latent form and takes milk-fever she is sure to die. I sometimes empty the rectum and bladder, and this sometimes gives the animal relief. I also sheet up the animal well, as there is a tendency to a subnormal temperature. I have tried stimulating embrocations along the spine when the cow was long in getting up, and when symptoms of paralysis are shown I prescribe doses of nux vomica. A great deal of success lies in the manner in which the cow is nursed. I give the attendants orders to turn the animal every four hours or so, but sometimes they neglect to do this very necessary part of the treatment, with the result that the cow gets her knees, hocks, etc., bruised, and then when she wants to rise she cannot, because of the sores and bruised condition of the muscles. There has been more success in the treatment of this disease in late years than before.

Now, gentlemen, I think I have taken up your time sufficiently, and I will come to a conclusion by saying that the lot of a country veterinary surgeon has its bright side as well as its dark side. He generally gets his clothes more soiled and is generally not so stylishly got up as his city brother. We have, as a rule, more dirty work and smaller pay for it, and we must appear in harmony with our work.

I beg to thank you for your patient hearing, and I trust that my effort will be rewarded by the members all participating in a good discussion.—*Veterinary Journal*, July, 1902.

---

FORMIN AS AN INTESTINAL ANTISEPTIC. According to the experiments of Soebisch, formin (hexamethylene-tetramine) is superior to all other substances ordinarily employed to disinfect the intestinal tract. It is very soluble in water, and possesses an important advantage in being non-poisonous. The dose for a person is two grains daily.—*Bull. Gen. de Therapeut.*

HYPERTROPHY OF THE HEART. The subject, a pig, died suddenly while being driven to the slaughter-pen. At the autopsy, made by Schrader, the heart was found to be very much hypertrophied, the weight 770 grammes, exceeding by 500 grammes the weight of the normal heart of an animal of the same size. The pig, which weighed 200 pounds, presented no other abnormal peculiarity.—*Ibid.*

